# Phrenology

**Published:** 1824-03-01

**Authors:** 


I. *Tansactions of the Phrenological Society, instituted 22d of February*, 1820. Octavo, pp. 448, with five engravings. Edinburgh, 1824.

II. *Phrenological Journal, 2d December*, 1823. 8vo, pp. 184.

We took the liberty of directing the attention of our readers to the subject of Phrenology in our number for last March. Neither become disciples of Gall and Spurzheim, nor professing hostility to their doctrine, we felt it impossible to abstain any longer from mentioning in our pages a subject which seemed to be gaining considerable ground in this country, of the truth of which many men of excellent understanding did not hesitate to declare themselves fully convinced, in support of which books of no little merit were appearing, and, above all, for the cultivation of which a society was actually established in Edinburgh and in America.

The former society has now sent forth into the world a volume of its Transactions, containing no fewer than 448 closely printed octavo pages; and we now purpose to lay its contents before our readers. No apology can be required. The omission would be a gross dereliction of duty. After the heavy fire which was opened upon phrenology a few years ago, it might well have been supposed completely demolished. But, no. When the smoke had rolled off, and the scene again become light, no desolation was discernible; there stood Phrenology, in all her original brightness, not a hair of her head injured, whole and undaunted; and, more than this, a band of doughty champions were found to have silently collected around her. The number of these has continued to increase. The society of Edinburgh consists of 86 members. Of these, nineteen appear, from having their names only announced, to be private gentlemen, and Sir George M'Kenzie, Bart. is at their head; nineteen are in the law; thirteen physicians; eleven surgeons; four ministers of the Scotch kirk; two colonels; three captains; five artists; the rest are civil engineers, bankers, merchants, booksellers, accomptants, and schoolmasters. There are members of both Oxford and Cambridge, fellows of the Royal Society of both London and Edinburgh, physicians and surgeons of great medical establishments, a professor of the University of Transylvania, and another of Berlin.

Men of such various situations and habits, for the most part necessarily of cultivated understanding and accustomed both to reflexion and independence of thought, all associated for the promotion of an object unconnected with religion or politics, (on either of which the wisest will grow extravagant,) and having no possible interest to serve, cannot but be supposed to have at least plausible reasons for their conviction; and we shall therefore be at some pains to present a faithful sketch of each paper in the volume.

The first is a *Preliminary Dissertation on the Progress and Application of Phrenology*, by Mr. George Combe, the well-known author of the Phrenological Essays. A short biographical notice is given of Drs. Gall and Spurzheim, and the mode in which the discovery of phrenology was made, is fully pointed out. It has been very incorrectly asserted, that the scheme of faculties in their system was drawn out by fancy, and the head afterwards divided into compartments corresponding with the list. Now the truth is, that Dr. Gall made his discoveries by noticing particular talents and dispositions to be of great strength in certain individuals, and particular parts of the head to be proportionally large. He thus discovered faculty after faculty, without knowing what was to come next.

"The method of enquiry pursued by Dr. Gall, having for its object the discovery of the relation betwixt the mental powers and their organs, possessed advantages peculiar to itself, and was free from the objections to which the methods of the physiologists and metaphysicians were liable. He attended to facts presented to his senses and understanding, and was thus led to a knowledge of a relation existing in nature, betwixt particular portions of the brain and particular faculties of the mind. However short, therefore, of complete success, his enquiries may appear to some at present to be, it is indisputable that his followers are in the road which leads to truth. His mode of philosophising has nothing in common with the formation of an hypothesis; and so far from a disposition to invent a theory being conspicuous, there appears, in the disjointed items of information which he at first presented to the public, a want of even an ordinary regard for systematic arrangement. His only object seems to have been, to furnish a candid and uncoloured statement of the facts in nature, which he observed; leaving their value to be ascertained by time and farther investigation." "But what followed? As soon as observation had brought to light the great body of the facts, and the functions of the faculties had been comtemplated with a philosophical eye, a system of the philosophy of the human mind presented itself, almost spontaneously, to view, (in the words of Mr. Abernethy) 'not like others presented to us, which appear in comparison but as mere diagrams, the result of study and imagination; whilst this seems like a portrait from life by masterly hands.' When the work of discovery had proceeded a certain length, the facts appeared to be connected by relations which it was impossible sooner to perceive; and system and arrangement at length suggested themselves where disorder only had previously reigned. Hence, it was not till after phrenology had been cultivated for several years, that its real nature and utility were discovered, and it was only then that its form became systematic. Hence, also, its character and its name changed as it proceeded; and from a mere species of physiognomy, it has become a science capable of the most useful and interesting applications. If, from the beginning, the discoveries of Drs. Gall and Spurzheim had assumed the aspect of a regular and polished science, and been moulded into complete accordance with the prevailing doctrines of the times, it would have been clear that they were theorizing; for a beautiful and perfect system could not arise at once out of the observation of facts. The history of discovery in chemistry and the other experimental sciences, shews that facts in nature may, for a time, appear, not only isolated and uninteresting, but absolutely inconsistent and perplexing, until future discoveries shall have linked them into the chain of connexion, and exhibited them in all the beauty and importance of essential parts in a system of truth. The very fact, therefore, that out of this mass of incoherence and apparent inconsistency, there has at last arisen a system of philosophy deserving of admiration, creates a strong presumption that it is founded in nature."

The advantages which must accrue to the philosophy of the human mind from phrenology are next stated, and the origin of the Edinburgh Phrenological Society is detailed, and the paper concludes with some observations on the nature of the evidence upon which phrenology rests.

"The proposition, that a certain mental power is connected, by nature, with a certain portion of the brain, relates to physical science, and can be proved or disproved only by observation. The province of reason, in such a case, is to compare the phenomena observed, to discover their relations, and to draw from them just conclusions; but not to determine, before-hand, whether the appearances alleged can, or cannot, exist in nature. Those persons, therefore, who attack the doctrines by mere arguments, whether founded on analogy or on previous opinions, only render complicated a question which in itself is simple; for if the facts alleged by the phrenologists really exist, all reasonings which go to contradict them must, of necessity, be defective in premises, or unsound in deduction; and if they do not exist, the whole fabric of the system will fall to the ground, without the aid of any other objection.

"The next consideration is the mode of bringing forward the evidence by which the science is supported.

"Delicacy to individuals stands opposed to the public statement of many interesting cases in favor of the doctrines. For my own part, I have been permitted minutely to examine the heads of several hundred persons, in different ranks of life, many of whom were well known by their talents as authors, preachers, public speakers, artists, &c. and the correspondence between their mental qualities and cerebral development was such as carried irresistible conviction to my own mind, that phrenology is a correct exposition of nature; but the confidence implied in permitting the examination imposes a restraint upon publication of the results, which no consideration will authorize me to disregard. It is on this account that phrenologists eagerly solicit those who wish to ascertain the truth of the system, to examine nature for their own satisfaction. Busts are sold, which indicate the situation of the organs, and books which describe their functions; and no one can have any difficulty in finding proper subjects for examination among individuals in his own circle, with whose talents and dispositions he is intimately acquainted. If, for example, two children are known, one of whom is extremely kind, and the other rash and precipitate, their heads may be placed in juxta-position, and their organs of cautiousness compared. The difference will be found so striking, that the most inexperienced observer may recognize it. In like manner, if, of two girls, one be particularly fond of admiration, and another be indifferent about her appearance, and regardless of the opinions of others, they may be placed in a similar situation, and a very palpable difference in their organs of love of approbation will be perceived. The degree of conviction resulting from observations of this kind, when repeated on a great variety of individuals, and in every diversity of circumstances, far surpasses that which can be produced by perusal of the most minute and authentic details of cases observed by others. By contemplating phenomena as they actually exist, the mind forms a judgment concerning the real nature of their relation to each other with a higher degree of certainty and satisfaction, than can be obtained by merely reading descriptions of their appearance, and of the order in which they occurred. In the former case, the enquirer satisfies himself by an examination of *all* the circumstances which he deems of importance; in the latter, he is apt to doubt that some material fact may have been overlooked, which, if stated, would alter the whole import of the experiment. These remarks are particularly applicable to phrenological investigations. By selecting for observation persons intimately known to himself, the enquirer will enjoy the means of estimating the real nature and extent of the talents and dispositions possessed, the actual appearance of the head, the effects of health, education, and of a variety of circumstances which he might imagine were not attended to in investigations conducted by others.

"The same restraints, however, do not oppose the publication of all cases bearing on the truth of phrenology. When individuals have rendered themselves conspicuous by their virtues or vices, by their talents or deficiencies of understanding, and when casts of their heads have, by their own consent, been placed in the hands of the public, or been properly acquired, there appears to be no impropriety in discussing openly the correspondence or discrepancy betwixt their cerebral development, and the known manifestations of their minds."

"Farther, when individuals have perished on the scaffold, and the Society has preserved authentic casts of their heads or skulls, there can be no impropriety in discussing, in the freest manner, the correspondence betwixt their mental manifestations and the development of their brains."

The second paper is entitled *Outlines of Phrenology*, and contains nothing new, but seems inserted for the purpose of affording a tolerable acquaintance with phrenology to any person, however uninformed upon the subject, who may happen to take up the book, and wish to understand its contents. A plate is appended containing four views of the head, with the organs delineated.

The third, by Dr. Poole, is *A View of some of Dr. Spurzheim's Lectures, as delivered at Edinburgh in the Winter of* 1816. Like the former it contains nothing new, but is probably inserted with the same object, and discusses some general questions. The account of the course opens in the following manner.

"Dr. Spurzheim commenced by apologizing for his faulty pronunciation and imperfect knowledge of the language in which he was about to deliver himself, and expressing his trust in the indulgent consideration of his hearers. But there seemed to be fewer demands on their favor or patience than he gave reasons for applauding him. Without the aid of notes, which a thorough acquaintance with his subject, and the habit of public speaking, had rendered unnecessary, he never failed to express himself in clear and forcible terms, free from embarrassment or affectation; and with the happy, but unassuming firmness of a man who believed that the importance and the truth of his discoveries formed a sufficient claim to attentive regard, and would redeem those trivial and adventitious errors which he might happen to commit in declaring them. His illustrations were copious and striking, the product of a mind matured in useful learning; but still more indebted to the long and varied exercise of native discernment on the concerns and characters of mankind. This gave peculiar value to his remarks, in the judgments of persons who had learned to distrust the theories of the schools, and to wait with some degree of patience, till an enlarged and well arranged series of facts should warrant a degree of confidence in the opinions which he delivered. 'Do not,' said he, 'believe any thing because I affirm it; nor, on the other hand, object to it merely because others have done so before. I may err, and others may err, but Nature is always true and constant. See and judge of her for yourselves.' In the same spirit, and with the same propriety, he distinguished between the facts which he meant to bring forward, as at all events deserving to be held important in the study of the human character and constitution, and those inferences which he or others had drawn from them. The former, he affirmed, *must* be admitted, and, accordingly, in regard to them, both he and his hearers were bound to identity of belief. But, with respect to the latter, especially in the present infancy of the science, some difference of sentiment might reasonably be anticipated, and certainly ought to be regarded without the admixture of angry passion, or a disposition to unprofitable cavilling."

We are then informed of the object of the science.

"The object of the science is not what it has often been asserted to be. No investigation into the *nature* of mind is even so much as attempted in it. We know no more of mind, as it is in itself, than we know of matter; and, accordingly, we must content ourselves with observations upon the properties of the one, and the manifestations of the other, as presented through the medium of our bodily organs. No argument, it is evident, is thence to be drawn as to the materiality of the mind. Such a question is never once agitated in the system; far less does the system afford any thing like an approach to the affirmative, as has sometimes, most erroneously, been imagined. The doctrine, it is acknowledged, may be abused; but in this respect, it does not differ from any other science, nor from any of the gifts of nature, all of which may be, and most of which have been productive of evil, through the depravity or the weakness of mankind.

"The object of the system, then, is the manifestation of the human mind, as dependent on, or connected with, organization. This dependence is assumed as essential, not to the existence, but to the manifestation of the mind, for we have no example of the operations or agency of the one, without the instrumentality of the other."

Several propositions are then stated. First, That the brain is allotted as the organ of mind,—"that without it no manifestation of mind has ever yet been known." Secondly, That a certain quantity of brain is required for the manifestation of the mind. Thirdly, That, *cœteris paribus*, the manifestations of the mind bear a proportion to the size of the brain; it being clearly asserted, that size is only *one* of the conditions on which the manifestations depend; for all will perceive, that the consistency of the brain, the temperament of the constitution, the degree of exercise of the talents, and other circumstances are likely to obtain a certain influence. Thus, although men remarkable for the versatility and general scope of their genius, have universally large brains, as in the familiar instance of Lord Bacon, and have the organ so developed as to give great fulness to the face and upper parts of the head, the converse is not maintained. We are reminded of Milton's description of the *fair large front* of our first parent; and of Shakespeare's expression, "*fore-heads villainously low*." The ridiculous doctrine of the affections being seated in the viscera of the chest and abdomen is then refuted. Bichat no where appears to so much disadvantage as when advocating this doctrine. The affections of the mind influence every part, and when the various viscera do not feel their influence, the affections must be powerless,—he who has not bowels of mercy, has them not, because he has no mercy in his soul: if the heart does not leap for joy, it does not, because the spirit is not enraptured. The cause is in the brain, the effect in the other parts.

The fourth proposition is, that the various parts of the brain perform various offices—that it consists of as many particular organs as there are primitive powers of mind. The arguments adduced in support of this proposition are, *first*, as Nature employs various means for various ends, and multiplies resources without limit, as occasion demands, organizing every plant suitably to its products, and every animal suitably to its circumstances and condition, and provides a particular organ for every animal function, and a distinct organ for each of the five senses, so analogy would lead us to presume, that all the mental functions and powers are accommodated with separate and properly adjusted organs. *Secondly*, That as the faculties and propensities of animals differ, so we must suppose their brains will differ, and so they actually do. *Thirdly*, The same individual manifests some properties and powers in a high degree, while others are scarcely discoverable in him, though quite natural, or frequently belonging to his species; one, for instance, having an excellent memory for words, but unable to carry on a process of reasoning; another displaying a genius for painting, but insensible to the charms of music; a third, excelling in mathematics, but devoid of all taste for poetry—facts apparently irreconcileable with the notion that one identical mass of brain presides over all the functions, but easily explicable on the supposition that the cerebral organs are as various as the functions. A precisely similar fact is the successive developement of the propensities and faculties. Lastly, the grand proof of the multiplicity of organs is the observation, that strength of faculty, sentiment, and propensity is accompanied by fulness of the brain at certain points, and consequent fulness of the cranium at the corresponding parts. Several considerations connected with this fact and absolutely necessary to be known, are fully described.

How satisfied phrenologists are that there is no evil in their doctrine, the following passage will shew. The writer concludes an account of a conversation he once had with Dr. Spurzheim on the subject of christianity in these words: "Of the character of Christ, he spoke with enthusiastic admiration, as the only perfect man;" and subjoins, "may I be allowed to add, that the objection to phrenology, as founded on materialism, and leading to infidelity, seems to me one of the most irrational and unjust allegations ever brought against any science. In reality, the perfect, explicit, unqualified correspondence of the doctrines of the system, with those writings of the sacred scriptures which relate to the character of man, is, to my mind, an incidental, but a powerful evidence of its truth, which, I confess, I have looked for in vain in the proud metaphysics of the schools."

To the fidelity of the following sketch of Dr. Spurzheim, we bear our humble testimony with pleasure.

"The vigour of his intellect refused no toil in the cause of science. The promptitude of his spirit and his zeal secured efficiency of judgment and patient enquiry on occasions which might have been thought trivial and unpromising by common observers. His serene, probably because his conscientious, reliance on the ultimate triumph of truth, supported him against the obstinacy of ignorance, and the malevolence of systematic error; and to these high endowments, so requisite to the character of a philosopher, especially when waging war with established creeds, he added a simplicity and gentleness of manners, which did not fail to conciliate regard, where his reasoning and his extensive information were urged seemingly in vain."

The same serenity, the same patience of opposition, and eager but dispassionate desire of discovering and supporting truth, and appearance of genuine philanthropy, we must say pervades all the papers in this volume, and we cannot do better than conclude with the following quotation.

"The prostitution of talents, which we have all of us had occasion to witness, in the opposition made to phrenology, and the frequent exposure of unhappy defects and bad habits, volunteered by some of its enemies, while they neither affect its foundation, nor ruffle the temper of its disciples, are productive of essential, but it must be allowed, very painful advantages; they furnish ample materials for the confirmation of its truth, and no less ample motives and opportunities for the exercise of that forbearance and compassion, which it is one of its excellencies imperatively to inculcate."

The fourth paper is by Mr. Scott, *on the Functions of Combativeness, Destructiveness, and Secretiveness—with illustrations of the Effects of different Degrees of their Endowment on the Character of Individuals.*

Our readers are no doubt aware that phrenologists divide the faculties or powers of the mind into three orders—those of intellect, sentiments, and propensities. Mr. Scott arranges the latter in three kinds—those which excite to action—those which restrain from it, and those which keep us in a permanent course of conduct—1. Amativeness, Combativeness, Destructiveness, Constructiveness and Acquisitiveness: 2. Secretiveness and Cautiousness; 3. Philoprogenitiveness and Inhabitiveness or Concentrativeness. The first he compares to the *steam* in an engine, the second to *the condensers and safety valves*, and the third to the *fly* which keeps the motion equable and steady.

*Combativeness* is not confined to the act of fighting, but is a blind impulse delighting in opposition for its own sake, a restless spirit of contention without end or object, but, under the direction of the higher powers, it gives boldness and force to the character, and enables these to act with energy and effect.

"While some individuals are so devoid of it as almost to run away from a Barbary hen, if her feathers turn back with any show of resistance, others are never so happy as when engaged in a vigorous contest."

Great arguers are well endowed with it, "and confute, change sides, and still confute." The Irish exhibit the beau ideal of combativeness; so natural is uproar to them, that a gentleman who understood them thoroughly, being asked how the people were disposed at the fair, replied that "all was peace and quiet, for he left them all fighting."

*Destructiveness* implies a desire not only of vanquishing our adversary, but of destroying him. The bloody games of the Romans illustrate this propensity.

"Were it not duly restrained, the earth would, as in the days before the flood, be, indeed, filled with violence; but it is restrained both by resistance from without, and by cautiousness, benevolence, and other superior sentiments within. It proceeds to the last extremity of murder, only when these restraints are too weak, or when they are overborne by a sudden excess of the destructive propensity, too strong to be resisted."

United with conscientiousness and benevolence, it never proceeds beyond virtuous indignation, and it is this faculty which appears to the writer to give the character its greatest energy and power.

The faculty of *secretiveness* is well described, and all its workings most ingeniously unfolded, and illustrated, like those of the two preceding, with great richness of quotation from the poets. But there is before us too much for us to indulge ourselves in any farther account, and we are not quite sure that the writer will be deemed equally satisfactory in discussing this faculty, as ingenious and elegant.

V. *On the Effects of Injuries of the Brain upon the Manifestations of the Mind.* By Mr. Andrew Combe.

We do not know that any greater nonsense has of late years been talked, than what we have heard from certain persons respecting the independence of the mind upon the brain. We are desired to believe that very large portions of the brain may be destroyed without the slightest impairment of the faculties; that all the brain, except about the bulk of an egg, may be destroyed, and the patient (if such he can be called) remain perfectly well, and a writer in a distinguished northern review, assured us, that he was never conscious of thinking with his head. He therefore, at any rate, could not be offended at any one refusing to consider him a *wise-head*. In their eagerness to prove the distinctness of mind from matter, they seem to have forgotten that the *facts*, which they adduce, prove far too much—prove not merely, that the mind and brain are distinct, but that the mind is unconnected with the brain—that the state of the brain has not the smallest influence upon the manifestation of the mind. Others have not proceeded to this length, but contented themselves with asserting, that a part of the brain may be destroyed, and yet the person remain in full enjoyment of his intellectual faculties, and that every part has been destroyed without the least deficiency of the faculty, which, according to Gall and Spurzheim, is corresponding.

The writer of the present very able paper, urges that the cases quoted were related merely for surgical purposes, and that, consequently, the observations upon the state of the mind were made without care, and the statements in regard to it are most unsatisfactory. "The senses were retained to the last; all the faculties remained entire; there was no deficiency in any intellectual faculty; no single power of the mind was impaired:" such is the information afforded us. In truth, we are ashamed of the logic which infers the full possession of intellect and feelings, from the ability of a patient to recognize his friends and answer plain questions. It reminds us of similar accounts of individuals exhausted by disease or age, "retaining their faculties to the last," as though they could investigate an abstruse point, or conduct an affair that required great sagacity and prompt energy.

In the volume of the Medico Chirurgical Transactions, just published, is an account of an infant that, to the dismay of the accoucheur, sucked, and cried, and kicked for some days after it came into the world, notwithstanding he had done his best to destroy it, by breaking up the whole of the two hemispheres of the brain before it came to light, into a pultaceous mass, and removed at least four ounces. Had the little fellow not unluckily died at last of hæmorrhage, he would no doubt have been able to learn to talk French and Latin, and sing Rossini's airs as well as other people, and take lessons in fencing and dancing, as well as to kick.

But not only are the assertions chargeable with general carelessness and vagueness, but the persons who made these observations were unacquainted with the faculties according to the system of Gall and Spurzheim, and consequently incapable of knowing in what way, relatively to the system of these philosophers, the mind was affected: not knowing, for example, that there was a principle of cautiousness, they would not think of enquiring whether the person was less cautious than before the accident.

"Finding none of the faculties which he calls attention, perception, memory, or investigation at all impaired, he, with great confidence, concludes, that the part in question *cannot* be the organ of cautiousness, and so satisfied is he with his own reasoning, that he laughs at those who do not see its cogency as clearly as he does himself. On any other subject, this mode of reasoning would be looked upon as proceeding from a very blameable and lamentable degree of ignorance, especially on the part of any one who comes forward uncalled for to the attack; but as directed against phrenology, it is looked upon by many as satisfactory, and philosophical in a high degree."

Further; without the knowledge of the situation, form, and direction of fibre of the several organs, of which the brain is composed, any observations must be imperfect. Now "nine tenths of the cases that are quoted, occurred long before the organs were discovered, and the remaining tenth, I believe, were observed in ignorance of the discovery."

Still further; the organs are supposed to reside not merely at the surface of the brain, but to extend into its substance; the loss of a portion of brain may therefore be only the loss of an exterior portion of an organ.

The doubleness of the organs of the brain also explains how one side might be seriously injured, and yet the functions be carried on entirely by the other—as urine is often secreted in due abundance after the destruction of one kidney.

"It will be seen from an attentive perusal of the cases quoted, that not a single instance is to be found, in which the destruction of both organs occurred, while the alledged manifestations of the mind existed."

Be it also remembered, that not only all the organs are double, according to phrenologists, but that "the organs of the intellectual faculties constitute so small a portion of the brain, as to leave nearly two thirds of the whole mass to be destroyed on *both* sides, without necessarily interfering with the intellect."

"It has never once been taken notice of by the opponents, that while they confine their attention to the state of the *intellectual faculties alone*, in all cases of wounded brain, the organs of these faculties in the new system, constitute not more than one third of the whole cerebral mass. The other two thirds constitute the organs of the sentiments and propensities, which are never enquired into, as not being conceived to have any thing to do with the brain."

With two exceptions, the remaining papers are all statements of facts illustrative of the doctrines of phrenology.

The first contains cases of deficiency in the power of perceiving colors. That some persons, whose sight is in all other respects excellent, cannot distinguish between certain colours, is well known in medicine. In the 7th vol. of the Medico-Chirurgical Transactions, an example is related of a family in this predicament: one, who was in the navy, purchased a blue jacket and waistcoat, and red breeches *to match;* and his grandfather, would mistake a cucumber for a lobster, and a piece of red sealing-wax for a leek. Phrenologists have fixed upon a certain portion of the eye-brow as the situation of the portion of brain in which resides the faculty of perceiving colours. Dr. Butter, of Plymouth, relates an example of the deficiency of this faculty, accompanied by a remarkable flatness of the orbitary ridge, in the spot assigned to the organ of colour. Another example, attended by a small development of the organ of colour, is, also, given by Mr. George Combe. The subject of this relation, Mr. Milne of Edinburgh, was placed in the Society, by the side of Mr. Gibson, a miniature painter, in whom the faculty was strong and the organ fully developed. A handkerchief was placed over the face of each, and more than half a dozen gentlemen, strangers to both Mr. Milne and Mr. Gibson, named them respectively and correctly, by feeling their heads at the part indicated. Mr. Combe mentions a third coincidence of deficient power and organic development in Mr. Sloane of Leith.

A case is subjoined by Mr. Combe, of defective power in recollecting landscape, attended by a small development of the organ of *size;* while, in another person, possessed of the faculty in great strength, this organ was fully developed: and, on comparing their heads, the greatest difference which was discoverable, existed between these two organs. This is an admirable method of learning the office of parts: by placing two individuals of great diversity in the degree of any one power, side by side, a great discrepancy in the proper organ of the power cannot but be detected.

The sixth paper, is a "*Notice of a Case in which the Patient suddenly forgot the Use of spoken and written Languages,*" by Mr. Alexander Hood. Among extraordinary cases of mental affection, none are more striking than those of persons whose intellect has been in all respects sound, except that they forget words and the meaning of them. This is an instance of that kind, and its importance arises from the circumstance of the disease having appeared to attack the part which is called, by phrenologists, the organ of language, for the patient had been subject to severe headaches, always referable to the sockets of the eyes and to the eye-brows.

The seventh is entitled, "*Remarks on the Cerebral Development of King Robert Bruce, compared with his Character as*

*appearing from History*," by Mr. William Scott. How phrenologists got a sight of the undoubted head of Bruce, we have no room to tell; nor to do any thing like justice to this masterly and beautiful paper. We believe our best plan will be to copy the statement of his majesty's development.

1. Amativeness—full.
2. Philoprogenitiveness—large.
3. Concentrativeness—rather full.
4. Adhesiveness—large.
5. Combativeness—large.
6. Destructiveness—large.
7. Constructiveness—rather small
8. Acquisitiveness—moderate.
9. Secretiveness—very large.
10. Self-esteem—rather large.
11. Love of approbation—large.
12. Cautiousness—large.
13. Benevolence—rather small.
14. Veneration—large.
15. Hope—large.
16. Ideality—moderate.
17. Conscientiousness—small.
18. Firmness—very large.
19. Individuality, lower—large.
    ———— upper—full.
20. Form—large.
21. Size—large.
22. Weight—uncertain.
23. Colouring—moderate.
24. Order—moderate.
25. Time—uncertain.
26. Number—uncertain.
27. Tune—moderate.
28. Language—uncertain.
29. Comparison—moderate.
30. Causality—moderate.
31. Wit—rather small.
32. Imitation—moderate.
33. Wonder—moderate.

The character of this development is one of great energy and power. From the full endowment of amativeness, philoprogenitiveness, and adhesiveness, the subject must have been a kind husband and father, and disposed to enjoy the pleasures of friendship and domestic life. The large combativeness and destructiveness indicate warmth of temper and prowess in battle. Bruce's cautiousness and secretiveness appeared in the general prudence of his measures, and his frequent employment of stratagem in attacking his enemies. His full share of self-esteem and love of approbation, joined to some portion of ideality, account for his high spirit of chivalry and his love of glory. His large firmness and hope were evinced in his perseverance in exertion, and his patience in distress. The latter was so remarkable, that Boece thinks, it could only have "cummin by miracle and grace of God."

Owing to his moderate acquisitiveness, we nowhere find him guilty of any mean or sordid action: of his veneration, he gave many unquestionable proofs, in the regard which he paid to the religion, and even the superstitions, of his time; and the last act of his life, was to order his heart to be conveyed to the Holy Land. His reflecting faculties were but poor, but in the times in which he flourished, and the situation which he held, the intellect of a Bacon would have served him in little stead. The age was barbarous; the Scots lived upon flesh in a half raw state, merely sodden in skins, and wore buskins of untanned leather; they were not superior to the Cossacks of our day. The duties of a monarch were little more than "to go in and out before the people, and to fight their battles." The development of his sentiments and propensities, with his large individuality, which must have given him a quick observation and a tenacious memory, fully account for his renown. The events of his life are all sketched out, and his conduct proved to be in perfect uniformity with his phrenological character.

The eighth paper is a "*Report upon the Cast of Miss Clara Fisher*," by Mr. George Combe. The extraordinary mental powers of this young female are well known. Her head is of prodigious size relatively to her age and stature. Phrenologists affirm that a small head never possesses energy. To represent a character well, it is indispensable that the actor possess a high endowment of those powers or feelings which distinguish the character. An actor possessing liitle combativeness and detructiveness, could not personate the fiery Coriolanus with effect. Hence, to constitute an accomplished actor, capable of sustaining a variety of parts, a general full endowment of the mental organs is required. *Imitation* is supposed to give the power of assuming character when the particular feelings enable us to enter into them; and secretiveness that of suppressing the natural character; and these two, perhaps aided by others, are regarded by Mr. Combe as general powers required in an actor. In regard to her excellent representation of Richard, Mr. C. says—

"The high and full forehead gives her the intellectual energy of that character. The immense love of approbation, firmness, and cautiousness, enable her to feel and to express the ambition, the determination, the coolness of the tyrant. The large secretiveness supplies the cunning; and the (very large) combativeness, and (full) destructiveness, the fire, and also the genuine obduracy of sentiments so characteristic of Richard; while ideality throws the colouring of poetry over the whole representation."

The ninth paper is the "*Case of J. G. aged* 10 *years*," by Mr. David Bridges, Jun.

J. G. was a lad picked up starving in the highway by a benevolent lady. His head was shown to Mr. George Combe without any intimation of his character or circumstances. Among other things, Mr. Combe reported that the organs of language, comparison, and wit, were full; of size, imitation, and ideality large; and of form and causality very large: that the organ of combativeness and acquisitiveness were full, of destructiveness large, and of cautiousness and secretiveness very large, while that of conscientiousness was small. He therefore stated, as his inferences, "that the boy would show uncommon penetration and considerable scope of mind;"

and as the weakest organ in the whole head was that of conscientiousness, while secretiveness was very large, there would be "a tendency to duplicity and finesse;" also, "that from his acquisitiveness being full, his honesty might be questionable; and that from combativeness being full and destructiveness large, and love of approbation moderate, the dispositions would probably be low and grovelling, and the temper hot."

Captain Davidson, who had taken the boy to Mr. Combe, then mentioned that the boy—

"Pretended he came from Glasgow, but that he was such a complete liar, thief, and swindler, that it was impossible to discover what his true history was; that he preferred sleeping in a dog-kennel or out-house, skulking like a fox, to sleeping in a comfortable bed in the dwelling house; that he appeared and disappeared nobody knew how, and even kind treatment did not render him social."

Mr. Combe gave Captain Davidson a note of the development, with some observations which concluded thus:—

"This subject is a fair one for education to do its utmost upon. Nature has given faculties susceptible of education, and she has left great wants to be supplied. If left to his natural tendencies, he will probably turn out a very first-rate swindler; if well educated, he may get through life without crime, and even with credit for his intellectual powers; but he will, with difficulty, be made amiable, sincere, and worthy of confidence."

We have next a letter from Mr. David Waddell, the tutor in Mrs. Cockburn's family, giving an account of the lad's conduct during the time that gentleman had the care of the boy's education. Mr. Waddell "feels no hesitation in declaring, that the leading features of his character correspond, in the most striking manner, with the development described in Mr. Combe's report."

The fellow pilfered every thing he could get at, opening trunks and drawers almost every day, and hiding the articles in his bed. His schemes were devised with a cunning, and executed with a caution, far beyond his years; and when he happened to be detected, he always absconded, and frequently eluded the keenest and strictest search.

The greatest pains were taken with him. It was intended to place him with Mr. James Milne, a brass-founder in Edinburgh, to learn the trade; and he was previously sent to a public school in Leith Wynd, and boarded with Mr. Retson, a person in the employment of Mr. Milne. A melancholy account of the boy's misconduct is given in a very long letter from Mr. Retson, and at last the boy decamped with his Sunday clothes, and eighteen-pence stolen from Mr. Retson's pocket, and has never since been heard of. The whole is an argument, undoubtedly, of the truth and use of phrenology.

The tenth paper is by Mr. George Combe, "*On inferring natural Dispositions and Talents from Development of Brain.*"

The custom of phrenologists to set down the development of every particular organ of the heads sent them for examination, and to infer from the absolute and relative size of each the talents and character of the individual, has been represented as mere quackery, near akin to fortune-telling. Mr. C. remarks that there is no quackery in the business: that if in a head the organs of intellect and moral sentiment greatly preponderate in size over those of the animal propensities, and the mental powers do correspond with the dimensions of the organs, the tendency of the mind will be strongest towards moral and intellectual pursuits, and feeblest, comparatively, in the range of animal desire. This inference is philosophical, and appears empirical only to those who disbelieve the facts—the principles on which it is founded; and the objector is unphilosophical till he disproves the truth of the principles.

Education, and all external circumstances, it will be said, exert an immence influence upon the character, and therefore the inferences from cerebral development, however indicative of the native strength of the faculties this may be, must be liable to immense error.

"The answer to this remark is simple. The phrenologist in no case ventures to predicate, from the mere development, any thing more than simple natural talents and dispositions; and in every instance, where a sketch, resembling that of actual character, has been given, previous information has been afforded of the age, sphere of life, and education, of the individual in question; and the conclusions have consisted of an estimate of the effects of these extensive causes operating upon, and modifying, the direction of the original powers."

When, however, the natural dispositions and talents are very decided, as in Shakspeare, Burns, and Buonaparte, they predominate over, and even command, events and circumstances; and a phrenologist, on viewing the heads of these mighty ones, might predicate—

"Quite philosophically, that Shakespeare possessed immense energy, an unbounded scope of fancy, and a very great talent for observation; that Burns was gifted with much manly, yet tender and simple feeling; and that Buonaparte had received at his birth an endowment of prodigious energy, joined with insatiable ambition and great intellectual power."

Where the organs of the animal propensities, moral sentiments, and intellectual powers, exist merely in equilibrio, education and circumstances are the most operative, because individual powers may be cultivated into a comparatively high state of activity. But when there is a great deficiency, or great development among the organs, education will have much ado in its efforts to raise those that are low, and depress those that are highly vigorous. In the former case the phrenologist would declare the great capability of the individual to take his character from circumstances, and would not draw his inferences till fully informed of these; while in the latter his inferences would be prompt and bold.

We are presented first with the development and a sketch of the character of a deceased Baptist minister. The surgeon who attended him and knew his character thoroughly, bore testimony to the accuracy of the phrenologists. Some remarks follow, explaining how the inferences were drawn from the development. Many organs act in opposition to each other, and it is by noting the relative as well as positive development,—by calculating the opposition, co-operation, and compensation of the different organs, that the inferences must be deduced. A remarkable fact in this case was, that the organs of cautiousness were very large, and the gentleman was remarkable for his circumspection, till attacked by his disease, which lasted six years, when his character quite changed in this respect, and after death those parts of the brain which Gall calls the organs of prudence and circumspection were found pulpy through disease.

We have next reports upon the development of the heads of several criminals.

The first report is by Mr. Robert Buchanan, on the development of James Gordon, executed at Dumfries, 6th June, 1821, for the murder of John Elliot, a pedlar boy. Gordon was a wandering Irishman, and, meeting with the poor boy, travelled with him a few days, and murdered him by striking his head with a clog bound round with iron, for the sake of his pedlar's box. After the trial, Gordon behaved very indecorously in court when sentence was pronounced upon him; but, at his execution, he prayed for himself and his judges, and acknowledged the justice of his punishment. *Combativeness* was found full; *destructiveness* very large; *conscientiousness* small; *benevolence* moderate; and all the organs of intellect small. The development of all the other organs is presented to the reader, and inferences drawn from the whole, by which it appears that the conduct of the unhappy man, throughout the business, perfectly coincided with his organization.

The second is on the case of John Bellingham, the assassin of Mr. Percival, by Sir George Stewart Mackenzie, Bart.

The circumstances of the assassination of Mr. Percival are well known. Bellingham was engaged in mercantile transactions, and, through the failure of a man with whom he was connected, became unable to fulfil contracts which he had formed, to a large amount, with merchants of Hull, who threw him into prison. Being of a violent temper and infatuated imagination, he viewed his imprisonment, not as an effect of his own imprudence, or as the natural consequence of one of those calamities to which human life is liable, but as a gross injustice; and published a pamphlet against the merchants, full of virulent invective, sarcasm, and ridicule. After regaining his liberty, he entered into complicated and extensive speculations, which involved him in more difficulties than before. Convinced, on this occasion also, that he had not to blame himself, and that he should obtain redress for his ideal injuries if he made them known, he memorialized Government again and again, and was put under confinement to restrain his turbulent proceedings. This new injury made him apply to the British minister at Archangel, but in vain; and after returning to England and marrying, though repeatedly told that Government could not interfere in his affairs, he continued to urge his unreasonable demands. He addressed to every member of Parliament a letter which is published in the Transactions with the answers of the Secretary of State and one to the magistrate at Bow Street,—an application which, no less than the idea of injury, and of right to redress from Government, proves the want of reflecting power, or of absolute insanity; as the police office could not, by any stretch of office, interfere with His Majesty's ministers. He then madly determined to murder some one or more of the members of Government, as though they could be responsible, individually or collectively, in such a case; and chose such a place and time for the deed, as no man of sound mind would have thought of, and instantly that it was over, sat down by the fire and avowed it. He professed repentance for all his sins, and often prayed fervently, but never could be brought to believe he had done wrong in shooting Mr. Percival. Sir George argues powerfully that Bellingham should have been considered a madman; and Dr. Spurzheim, on taking up his skull, without knowing whose it was, said it must be the skull of a murderer.

The whole head was very small, and the largest parts were the sides and occiput, the forehead being very low and narrow,—shewing deficient intellect and violent passions. The statement of the organs mentions *compassion* considerably small; (forehead slopes gradually;) *benevolence* very small; *conscientiousness*, small; *cautiousness*, moderate; *firmness*, large; *self-esteem*, large; *love of approbation*, full; *acqui-*

*sitiveness*, large; *combativeness*, very large; *destructiveness*, very large.

"Probably there never was a development and character more truly coincident than those of Bellingham. The weakness of his understanding is indicated, both by the smallness of the whole head, and by the small proportion of the forehead. It is obvious that his ideas of his own importance were high; while his *love of approbation* must have rendered him more liable to disappointment. His *acquisitiveness*, want of *benevolence*, and *self-esteem*, must have concentrated his whole thoughts in self. His *cautiousness* being moderate, he had little prudential feeling. His great courage and *firmness* led to his persevering and harrassing complaints, which he could not, from want of intellect, perceive to be unreasonable; and his full organ of *hope*, indicates a strong excitement to perseverance. The very little conscientiousness he had, was confined entirely to his own case, and perverted. His very large organ of destructiveness, besides indicating a violent temper, and a disregard to the feelings of others, inspired him with an impatient activity, and led him to resolve upon the horrid deed which he committed. Whatever pity he might have expressed for the family of his victim, his pretensions to that feeling are flatly contradicted, by the pains he took to state that he did not repent of what he had done, as well as by the smallness of the organ of *benevolence*. On the whole, a case more favourable for phrenology can scarcely be imagined; and it is one, too, equally favourable for demonstrating how erroneous the law with respect to responsibility. To say with the Attorney-General in this case, that a man deficient in understanding, is, nevertheless, capable of distinguishing right from wrong, would be to say too much, without defining the limit between sanity and insanity, and which, probably, is not to be discovered. A man, without much intellect, may indeed possess, according to phrenology, that feeling which prompts just actions, and gives an aversion to such as are unjust. But setting phrenological knowledge aside, the opinion of the Attorney-General may be considered tantamount to the assertion, that idiots are responsible for their actions, idiots being no other than human beings deficient in understanding. But the case of the wretched Bellingham was more deplorable than that of an idiot; for, with a very deficient understanding, he had opposed to it all the lower propensities of human nature, in a high degree of activity."

Mr. George Combe furnishes the last report upon executed criminals,—observations on the cerebral development and dispositions and talents of Mary Macinnes.

This woman was the keeper of a brothel in Edinburgh, where, in a skuffle that had begun during her absence, she stabbed a man, after taking a knife deliberately from a knife-case on the dresser, and before a syllable had been said to her or any provocation offered by the party.

In the account of her development we find *amativeness*, very large, *adhesiveness*, large; *combativeness*, enormously large; *destructiveness*, large; *secretiveness*, large; love of *approbation*, large; *benevolence*, *veneration* and *hope*, full; *conscientiousness*, rather small, with a meagre development of the intellectual organs.

"The organs of the lower propensities are here very largely developed, while those of the intellect and moral sentiments are proportionally deficient. Her education was very limited. She could read in some degree, and she attempted also to write; but her penmanship was very imperfect. Her *love of approbation* was so strong, that she pretended to those attainments in a higher degree than she really possessed them. The organ of the amative propensity is very large for a female. It is combined with a very large combativeness, which gives boldness to the character, and is not proportionally restrained by *conscientiousness*, in the reflecting powers; and this combination sufficiently accounts for the line of life which she adopted, followed up by the commission of the crime for which she suffered."

The organ of *destructiveness* is large, and the size of this and of the organ of combativeness corresponds with her irritability. The murder was committed in a rage; she was universally allowed to be very passionate, and on the Monday before her execution, she evinced the most violent emotions of rage on being refused to see a person to whom she was attached. From the smallness of her benevolence and reflecting faculties, compared with destructiveness and combativeness, Mr. Combe explains why she at first viewed the murder with complacency, and never expressed any very keen remorse at it. Several curious circumstances correspond with the large size of the organ of *secretiveness*.

Her father's name was Macinnes, and he was a lime burner in Isla, but she persisted she was the daughter of an adjutant Mackinnon.

"Her sister visited her in jail, while under sentence of death, and in an agony of grief, entreated her to disabuse the public of this monstrous falsehood, and not to die with such a palpable and wicked lie on her lips; but Mary remained unmoved by her entreaties, and maintained her own story. The only indication that she ever gave of departing from it, was on her way to the scaffold. The Rev. Mr. Porteous then asked her, if she still adhered to her declaration respecting her parentage: she paused, made no answer, and changed the discourse to another topic. This, it may be observed, was equal to a confession of the falsehood of her statement; for while she anxiously reiterated the declarations of her innocence of the murder, it is not probable that she would have failed to support her former statement, if it had been true, by a renewed assertion upon this point also. During her confinement, she presented the most profound silence regarding the persons who had been connected with her in her previous life,"—and Mr. Thomson never recollected a prisoner making fewer confessions than she did.

On the other hand, *adhesiveness* and love of approbation were large, and *veneration*, *hope*, and *benevolence* full. Accordingly, it seems, she was an affectionate daughter, affording assistance to her parents, and visiting them from time to time: she kept them in ignorance, as to her occupation: she was kind to the sick poor of her neighbourhood. She was very superstitious, and looked for the royal pardon, founding her expectation on dreams and the imagined applications of the ladies of Edinburgh in her behalf. *Firmness* was largely developed, and the clergyman testifies that she evinced, at the time of execution, an extraordinary degree of fortitude. Her organs of the reflecting faculties were small, and the clergyman found it impossible by reasoning, illustration or explanation, to give her any tolerably distinct apprehension of the scripture doctrines: and he was satisfied that this arose not from defective education, but from natural deficiency. Her strength of attachment was shewn upon the scaffold. A person, whom she loved, had sent her a handkerchief, with his name in one corner, and half an orange, to eat at her execution, he having eaten the other half at the same hour, the preceding morning.

"She held the corner of the napkin in her mouth almost all the night preceding her execution, and even on the scaffold. When seated on the drop, the turnkey gave her the half orange. She took it out of his hand, and without the least symptom of fear, said, 'tell him (the object of her attachment) that I die perfectly satisfied that he has done all in his power for my life, and that I eat the orange as he desired me. May God bless him!' She seemed to forget eternity in the ardour of her attachment to earth."

Mr. Combe concludes with a few general reflections upon the difference of the heads of such characters and of amiable persons. The Rev. Mr. M. whose development and character were mentioned at the beginning of the paper, was the very reverse of poor Macinnes. In the words of Cowper,

His mind was tempered happily, and mixed
With such ingredients of good sense and taste
Of what is excellent in man.

"How opposite is the character of Macinnes! on comparing the two heads, you will perceive, that *a great mass of the brain in the head of Macinnes lies behind the ear towards the base of the skull; that the forehead is low, and the coronal surface narrow. In the Rev. Mr. M. on the other hand, by far the larger portion of the brain is situate before the ear; the forehead possesses a full development, and the coronal surface is highly expanded.* You are aware that the society possesses casts of nearly thirty murderers; and that in every one of them, without a single exception, a large development of the animal organs, in proportion to those of the intellect and moral sentiments, is found. These casts and skulls of murderers have been collected from Paris, London, Nottingham, York, Dumfries, Glasgow and Edinburgh, and the same type runs through the whole of them. On the other hand, the society possesses a variety of casts of the heads of virtuous individuals, and in all of them, an evident preponderance prevails in the development of the moral and intellectual organs, as in the case of the Rev. Mr. M. These are facts at which the superficial may smile; but they give pause to the philosopher, and lead him to very serious contemplations."

The tenth paper is *On the Modes of Studying the Natural Dispositions and Instincts of the Lower Animals,* by Mr. Andrew Carmichael.

The plan is,

"To form four columns, under the name of the animal. In the first column to insert all the habits, &c. of the animal recorded. In the second, to reduce these to such of the thirty three faculties of man, as they might most properly be ascribed to. In the third, to state whether the respective organs had been ascertained or not: and to leave the fourth for observations respecting the differences between the male and female, and for pointing out prominences supposed to be organs, the faculties of which have not yet been discovered."

The eleventh, is a *Phrenological Analysis of some of the Maxims of La Rochefaucault,* by Mr. George Combe. It is extremely good, but altogether insusceptible of a brief epitome.

The twelfth is by Mr. Andrew Combe, and entitled, *Observations on Dr. Barclay's Objections to Phrenology.*

In his compilation of all the wisdom and absurdities that men have written upon life and mind, Dr. Barclay condemns phrenology as visionary, and the object of the present paper is to examine the arguments, for he advances no facts, upon which his unqualified judgment is pronounced. Mr. A. C. complains that Dr. Barclay assumes that the facts have no foundation and then argues that the phrenological conclusions are inconsistent, not with fact, but with preconceived notions of his own, altogether foreign to the question.

Dr. B. asserts, that voluntary organs are not restricted to any specific modes of operation, and illustrates the assertion by the various acts which the hand can perform. The hand, Mr. A. C. truly replies, is not indeed restricted to specific *acts*, (it may do ten million things) but it is restricted to a specific mode of operation, namely, to muscular contraction and relaxation.

Dr. B. continues, "taking the hand, then, as a specimen of the works of nature and of animal structure, and thence, reasoning on the principles of analogy, with respect to the brain, ought we not to infer, that all the parts, of which it is composed, may also combine in a similar manner, and be concerned in every phenomenon which has been ascribed to it." To maintain this analogy, it is obvious that all the operations of the mind should be referrible to one principle, say to feeling or reflection, but not to two or more. In amputating a diseased limb from beneficence, or a sound limb from cruelty, the action of the muscles is the same, but the motives are opposite; and analogy, if correct, would lead us to infer, that the emotions of both cases were the same, and experienced by the same mental organs. Analogy, here, however, is against Dr. B. for there is as great difference between the feeling of benevolence and of cruelty, as between the sense of smell and of sound: and these have distinct organs.

Dr. B. next denies the existence of a plurality of organs in the brain, because no such things are observable, he says, on opening the head. The fact, however, remains the same,—that strength of certain faculties and feelings is commensurate with the size of particular parts of the brain. Moreover, whatever Dr. B. may not be able to discover, it is very certain, that any one, who attends *minutely* to the appearance of the brain, may distinguish the anterior, middle, and posterior lobes of the brain, even if seen separately; may discover great differences among the convolutions; and see the organs of benevolence, &c. developed in various proportions. In proof of the differences observable in the brain, we present our readers with the following account.

"On 1st Dec. 1818, when engaged in the first course, which he gave after his return to Paris, a brain was handed to Dr. Spurzheim, during lecture, with a request that he would say what characteristic dispositions it indicated; and he would then be informed to whom it had belonged, and how far he was correct." The brain had considerably changed its shape from lying in a flat dish. "He desired his auditors to remark the size of the cerebellum, and the great development of the posterior, and of part of the middle lobes of the brain, the convolutions of which were large and rounded, forming a contrast with the deficient size of the anterior lobes. The convolutions situated under the vertex, and towards the top of the head, belonging to the organs of *self-esteem* and *firmness* were also very large, while those of veneration and benevolence were small. These peculiarities were so well marked, that Dr. Spurzheim felt no difficulty in inferring from the large size of the cerebellum, that the individual would have des dispositions fortes à l'amour physique. From the large development of the convolutions belonging to the lower propensities, common to men and animals, and from the small endowment of intellect and moral sentiments or restraining powers, he inferred that 'his natural tendencies would not be towards virtue;' that he would be what is familiarly expressed in French by 'un mauvais sujet;' being a very comprehensive term for every variety of bad dispositions, and that 'he would be one to whom the law would be necessary as a guide;' but not knowing the circumstances in which he had been placed, he could not say what his actions might have been. At the conclusion of the lecture, a young man, an eléve interne of the Hôtel Dieu, came forward and said, that the brain was that of a suicide, who had died in that hospital, and that the dispositions inferred coincided perfectly with those manifested during life."

The man had been a soldier, and banished from Paris to Orleans, for a crime that he would never name, and for which he had been subjected to a peine infamante. From the news reaching Orleans, he got no business, and, suspecting his wife of having contributed to his banishment, he returned to Paris, resolved to kill her and then himself. In the attempt to do the former, he failed; and then plunged the knife deep into his own body, between the 7th and 8th ribs. During his stay at the Hotel Dieu, he showed the most determined obstinacy, abused every one, particularly his wife, and declared he should like to kill her. He tore away the dressings; opposed every prescription, and died. Dupuytren, in his clinical lectures, often laid great stress upon the mauvais moral of the man—that le malade s'impatientoit contre tous les remèdes, etoit très impèrieux, et donnoit un negatif directe aux prescriptions.

A person who has devoted but little attention to the appearance of the convolutions, may imagine that little difference is to be observed among them; but let any one examine a large number of brains with care, and he will discover very characteristic distinctions. He will find "that the anterior lobe of the brain uniformly presents convolutions different in appearance, direction, and size, from those of the middle lobe; while the latter, towards the coronal surface, uniformly presents convolutions differing in appearance and direction from those of the posterior lobe; and, *above all*, that the cerebellum, or organ of amativeness, is not only widely different in structure, but is separated by a strong membrane from all other organs, and can never be mistaken for any of them." "The carefnl inspection of a few brains will satisfy any one that such differences actually exist in the different parts of the brain, which, joined to the fact of the development of no one of these parts bearing any relation whatever to the development of others, would lead us to infer that each had a distinct function, even although we had not the analogy of other parts of the nervous system to support us."

The analogy alluded to, is the fact, which Mr. A. C. considers proved by Mr. C. Bell and Magendie, of nerves, similar in appearance, performing perfectly distinct functions.

When Dr. Barclay objects that an anatomist, on seeing an ear, eye, &c. instantly names its nature, functions, and situation, but that no such thing is possible in regard to the imagined organs of the brain: Mr. C. replies, that "the use, &c. of these parts is *previously learnt by observation*, and that, if Dr. Barclay were shewn an insulated branch of the fifth or ninth pair of nerves, for example, performing, as they are admitted to do, distinct functions, he would not easily say where it had been situated," how connected, or in what functions employed. As to the cerebellum, Dr. B. must be allowed by every one to be totally wrong: it can never be mistaken for any other cerebral organ; and if brains were as often seen as faces, there can be no doubt that the characteristic differences of its parts would long ago have been fully known.

Dr. Barclay next quizzes phrenologists for finding no organs at the base of the brain, but all upon the upper parts and sides, "where they can at all times be easily seen." Now, here is a proof that phrenologists ground their statements on fact—on what they observe only: had they fabricated their system, they would have announced it perfect at first,—they would, in all probability, have divided the *whole* brain into compartments, and the more concealed parts at the base would have answered their purpose the best, as giving the least opportunity for refutation. If no organs are yet stated to reside at the base, this is no argument that the assertions of phrenologists regarding other parts are false: the system may not be complete, but what is known, is not, *on that account*, the less true. In fact, we ourselves should not suppose that many organs of distinct faculties would ever be found there. The base is but a small portion, and many of its parts are obviously for mere connexion.

Dr. B. says, there is no visible part in the crown of the head, frontal bone, occiput, or temples, where organs do not present themselves, and that they avoid the central parts. Now, when Dr. B. wrote this, there was a blank left in all the plates and casts of the phrenological heads, marked by Dr. Spurzheim between Nos. 16 and 23; and Dr. Spurzheim always mentions, that he believes the organs are not confined to the surface, but extend from the pyramidal bundles of the medulla oblongata; and, as the ear is generally almost on a line with the upper part of the medulla oblongata, he proposes to estimate the length of a cerebral fibre, by drawing a line from the orifice of the ear to the circumference, in the direction of any particular organ. And if phrenologists had believed them to be confined to the surface, they might have owned themselves ignorant of the use of the central parts, without thereby giving any one a right to impugn their observations regarding those at the surface. If Dr. B. is singularly unhappy in these reasonings, he becomes absolutely ludicrous, when he grumbles because the infundibulum and four ventricles are not admitted into the number of organs. When he receives thunders of applause from an empty class-room, he may expect the holes and cavities of the brain to perform the offices of material organs.

Our readers may think Dr. B. sufficiently good, without aiming at any thing more ridiculous: but, it appears, he thinks otherwise, and actually raises another objection to the plurality of organs in the brain, because no muscular action is assigned to them!! The next time he writes, he will probably find fault that the brain is not allowed a side-pocket. The belief of a plurality of cerebral organs cannot do away with the belief of muscularity, if the brain, considered as a single organ, is shewn by Dr. B. to be muscular. The good doctor, from working so much upon muscles, seems to like nothing else—he would have

> Muscles in trees, muscles in running brooks,
> Muscles in stones, muscles in every thing.

The ostensible reason why he wishes phrenologists to give muscles to the brain is, that it may have instruments to execute its will. Now the brain has such instruments—all the muscles of the body, with the bones, ligaments, &c. are the ready ministers of its will—" where, says Dr. Barclay, are these corresponding executive organs ? to this no answer has been given, &c. The state is not much to be envied, when there are more to advise than to listen, and more to command than are willing to obey, in all well regulated fleets and armies, &c." There are not only some to listen, and some to obey, but more to listen and obey than to advise and command, precisely as in all well regulated fleets and armies. Phrenologists have satisfied themselves at present of about thirty-four cerebral organs, and, besides the six organs for hearing, vision, smell and taste, and the two hands for touch, and the whole body for feeling, all which are the mind's active informers, there are 220 *pairs* of muscles of voluntary motion, besides bones, ligaments and cartilages, all obviously essential to the performance of any act prompted by the will. Dr. Barclay's string of objections is really most unhappy—sent un peu l'apoplexie. Mr. A. Combe then proceeds to show that a variety of phenomena of both health and disease are explicable on the supposition of a plurality of organs in the brain, but not upon that of the brain being a single organ. These are 1st, the *successive* development of mental powers. The child is all alive to the existence, properties, and circumstances of surrounding objects; the adult is able to reason. In the child no sexual desire is felt: in the adult it forms a striking character of the mind. In truth, the brain experiences corresponding changes. The middle and lower parts of the forehead generally predominate in childhood, and the upper and lateral parts become more prominent in later life. We must confess that we have seen at Mr. Deville's, in the Strand, a striking example of the successive development of the parts of the brain correspondently with the development of the mind. There are two casts of a young man, one taken when he came of age, the other two years subsequently: the portion of head assigned to intellect —to causality and comparison, project in the second cast so much more than in the first, that no person can fail to detect the difference. Chaussier says, that the cerebellum (the supposed organ of sexual desire) forms $\frac{1}{17}$ of the encephalic mass in infancy; and from $\frac{1}{6}$ to $\frac{1}{8}$ of it in the adult. The growth of the whole brain, and the successive proportionate development of its different parts, for the first third of our existence, cannot be denied. Whatever certain foreign anatomists have asserted to the contrary, any one may satisfy himself of this, by taking a cast of a young person every year or two. We hope phrenologists will provide themselves with some sets of documents of this nature.

The 2d fact explicable only on the plurality of cerebral organs is the partial nature of genius: one person being endowed with one talent, a second with another, and possessing no excellence, perhaps being really deficient, in other respects. Were the brain a single organ, there could be no partial excellences.

3. The phenomena of dreaming, in which numerous emotions and numerous conceptions occur, succeeding each other without intellectual control. These harmonize with the notion of a variety of faculties and organs, some being active, and giving the ideas and feelings which constitute dreaming; while others are inactive which would control them and prevent the diorder that characterises the fancies of sleep. If the organ of mind were single, dreaming could not occur: the faculties should be all asleep or awake at the same time.

4. Partial idiotcy and partial insanity afford very strong arguments in favour of the plurality of organs. Some idiots can calculate well; others can sketch accurately: lunatics are frequently insane on some topics only. Were the brain a single organ, there would be no partial deficiencies.

5. Partial injuries of the brain are the last fact mentioned. If the whole brain be concerned in every thought and feeling, it is wonderful that a portion can be lost or injured, and all the processes of mind manifested with *equal success* as in health. In good truth, these are not manifested as before; they are partially impaired, if both sides are injured,—some are injured—and this proves the plurality and diversity of cerebral organs.

Mr. A. C. concludes with exposing the inconsistency and absurdity of many of Dr. Barclay's remarks, and certainly has answered the doctor in so powerful, so clear, and withal in so respectful and gentlemanlike a manner, that Dr. Barclay's truest friends will advise him to say no more upon the subject.*

XIII. The last paper is "*On the Phrenology of Hindostan,*" by Dr. George Murray Patterson. This highly interesting paper contains, we must allow, powerful arguments of the truth of phrenology. The character of the Hindoos is totally dissimilar from that of Europeans, and is well ascertained. Their cerebral development, we learn, is precisely correspondent, and the grounds of this assertion are an examination of not less than 3000 heads of the natives; and we need wonder no longer that a few Europeans manage a host of Hindoos.

The whole head is smaller, and size of brain is, *cœteris paribus*, a measure of mental energy. The development of the anterior parts of the frontal bone, the region of the knowing faculties, is less full. The knowing faculties are, in fact, less active; but the organic development, and the mental manifestation of individuality, number, and language, are the most considerable among these faculties. The Hindoo is very much alive to surrounding objects: he calculates single numbers quickly and correctly: his native language abounds in signs, which he easily remembers. The organs of *form*,

---

* We shall take this opportunity of noticing a strange objection of Dr. Barclay's to the use of the word *organization*. This, he says, signifies the circumstance or act of organizing; and the structure produced shall be termed an organism. Now the plain answer is, that the word *organization* is universally employed in both senses—all persons, when speaking of the business of organizing, say the organization of such a body requires so and so; and likewise, when speaking of a body, &c. already organized, call it an organization; and they do this, borne out by analogy as well as prevalent custom. Formation signifies the act of forming, and the thing formed; production the act of producing, and the thing produced; preparation, a word in daily use with anatomists, chemists, and apothecaries, signifies both the act of preparing and the thing that is prepared. We therefore trust that phrenologists and all physiologists will say organization whenever they think proper.

*locality*, and *time*, are weak: and when they wish to point our resemblances in form, they are obliged to assist their memory by keeping an object before their eyes; they have extreme difficulty in remembering where they have laid any thing, and are not addicted to travelling; their songs are destitute of melody, and are a mere monotony of recitation.

The organs of the reflecting faculties are small; but that of causality much smaller than the organ of comparison: they are fond of similitudes, but have no great power at pointing out differences. They are continually expressing surprise, so hard is it for them to account for any thing.

The organs of wit and imitation are small; they rarely laugh involuntarily, (the effect of wit in those who can taste it is irresistible laughter,) and few excel in mimicry. The organ of benevolence is small, and a Hindoo cannot easily be made to comprehend the meaning of a disinterested action; if you succeed he only considers the person who acts disinterestedly as a bura pag hul, a great ninny. The mildness of the Hindoo arises from the deficiency of the organs of combativeness and destructiveness.

The organ of veneration is very moderate, but larger in Brahmins, whose whole heads, we are told, (and the fact is very striking,) are better organized than in other casts.

Ideality is rather more than moderate, and their poetical effusions are highly florid and allegorical, though defective in moral sentiment.

The organs of self-esteem and love of approbation are very fully developed; and Mr. Hamilton says, that "there is scarcely a creature so wretched or so ignorant, but who, on this account, holds in the utmost contempt many persons in easy circumstances and respectable situations."

The organ of cautiousness is so uniformly very large, that the protuberance may be seen at a great distance; whence their natural timidity.

The organs of amativeness and philoprogenitiveness are large; the latter very large. Their swarms of children, polygamy, and frequent unnatural crimes, and extreme affection for their offspring, are well-known.

The organs of acquisitiveness and secretiveness are very large. Their lying, hypocritical, pilfering character is precisely what a phrenologist would expect.

The organs of constructiveness, combativeness, and destructiveness, are small.

In the division of Hindostan that has been subjected to the Mussulmans, the organization of the head is superior to that which is seen where the Hindoos remain in their purity. In provinces possessed by the British, and inhabited by many

Mussulmans the cerebral organization is so much inferior, and the manners so improved and civilized, that the Hindoos there seem a distinct variety of men. Cultivation and admixture of the English breeds will therefore do much for the people: the former only can be brought into full play, and its effects are slow, but they are certain.

We close our account of this volume by assuring our readers that it will make every phrenologist proud, that it will probably convert many unbelievers, and highly delight those who remain in their unbelief.

And here we thought our phrenological labours at an end, when, lo! there appears a more unexpected thing than a volume of Phrenological Tranasctions—positively the first number of a Phrenological Journal. We are not in the habit of giving an account of Journals, but on this extraordinary occasion we shall deviate from our habits, not however entering into much detail, because the book is cheap and may be readily procured.

The object of the work is, of course, to communicate all phrenological discoveries and novelties, and news about phrenologists and phrenological societies. It professes, likewise, to defend the favourite science against all attacks and side hits of periodical publications; to try characters in novels and poems, &c. on phrenological principles; and dissipate popular objections of all sorts. The introductory statement from which we gather this, contains a copious list of *railing* and *abuse*, *falsehoods* and *malignities*, *impertinencies* and *insolences*, *wretched jokes*, *indecencies*, *nastinesses*, and *brutalities*, from Blackwood's Magazine, the Edinburgh Review, &c. as a specimen of their mode of holding out sinners to public contempt.

The first article is a very humorous petition from the metaphysicians to the most profound, impenetrable, and mysterious powers—chaos, night, and dulness—for the suppression of phrenology.

The second is an answer to recent attacks on phrenology, and complains that phrenologists are not opposed by a disproval of their facts, but that these are at once ridiculed without examination, and wit and cunning (if such names are deserved by their attempts) employed at once to sweep away the whole.

"This is exactly of a piece with the attempts made in the sixteenth century, and even later, to ridicule and reason down the Copernican System. The wits and reasoners of those days, when they set themselves in array against the observers of nature, disdained to proceed by the slow methods of observation and induction. They at once denounced the notion of the Earth's motion round its axis as contrary to *common sense.* But we do not now consider the doctrine of the earth's rotation to be *contrary* to common sense, although common sense alone could never have discovered it.

"When we survey the solid foundation of facts,—innumerable, incontestible, well observed facts, upon which the conclusions of phrenology rest, we who have studied these, and who know what it is we trust in, can regard, with a degree of calm contempt, of which our adversaries can hardly form a notion, the prodigiously imbecile attempts which have been made to put the system down, either by what is considered Reasoning, or what has very erroneously been designated Wit. We certainly are not so unreasonable as to expect, that, on the first announcement of a new science such as this, founded on an extensive observation of facts, our assertions shall be admitted without examination. But we certainly have a right to complain of those who will neither believe us, nor examine for themselves.

"The facts observed by the phrenologists are got rid of in a very summary way. 'Who knows, *for example*,' asks one, 'whether, 'for every case brought forward by them, there may not be one of 'an opposite kind *kept* in the back ground?' To say nothing of the delicacy, the candour, or the justice of such a supposition, or rather such a charge (for it infers the utmost disingenuousness and even moral depravity in the phrenologists,) we shall merely remark, that it is utterly inadmissible in fair argument. We are not here in an Ossianic or a Rowleian controversy, respecting the authenticity of manuscripts which nobody ever saw, or the genuineness of traditionary poetry which nobody ever heard. The facts are as patent to all as the light of heaven, and within the reach of every, even the meanest, capacity. Nay farther, so very open have the phrenologists laid themselves to detection, that they have published to the world their methods of observation, and furnished every one with the means of making observations for himself, whereby he may be enabled to detect their errors, if they are in error, and to expose their quackery, if they are really quacks. It is certainly incumbent on those who deny the universality of the coincidences asserted, to prove the exceptions, and to produce instances to the contrary. They have been challenged to do so for at least ten years; and they have been challenged in vain. What is the legitimate inference to be drawn from this?—Plainly, *that they have no facts to produce;* and, considering the numbers, the weight, the abilities, and the zeal, of the opponents of phrenology,—the rank which they hold in science, and the interest they have to maintain their supremacy,—it is not too much to say, that, *had any such contradictory facts existed, they would have been produced before now.* To this we cannot imagine the shadow of an answer."—*Phrenological Journal, p.* 22.

To show that the science rests upon facts, the evidence of the existence of the propensity and organ of destructiveness is examined, and we fear it will prove but too destructive to the character for sound reasoning of those who think proper to disguise it. There are twenty very interesting pages of evidence.

The third and fourth articles contain some complaints against Dr. Barclay and Professor Jameson: we hope those gentlemen will exculpate themselves.

The fifth is an abstract of Mr. C. Bell's discoveries of different functions being performed by different nerves, from the Philosophical Transactions;—this gentleman appearing to have shown with respect to the nerves, as Gall did with respect to the brain, that different nerves have not the same offices, nor the same nerves various offices.

The sixth is a dialogue between a disciple of the old school and a phrenologist, displaying the manner in which people make up their minds that phrenology is all vanity and vexation of spirit.

The seventh is to show that Reil does not detract from Gall's merit, but, on the contrary, speaks of his discoveries in the highest terms. Professor Bischoff says that Reil declares "that he had found more in Gall's dissections of the brain than he thought any man would have been able to discover there during his whole life." The celebrated anatomist Loder also says, "I am ashamed and angry with myself for having, like others, during near thirty years, cut slices of brain as we cut cheese, and not have seen the forest on account of the number of the trees. But why be vexed and ashamed? The best way is to listen to truth, and learn what one is ignorant of. I say, like Reil, that I have found more than I thought a man could have discovered during his whole life."

The eighth is an allegory of a spider and a bee—the former staying at home, forming no acquaintance with the world, and producing nothing useful or agreeable, representing the metaphysicians; and the latter, roaming in the sunshine over all the beautiful productions of nature, and effecting a substantial and delicious nourishment, representing the phrenologists. The simplicity, correctness, and elegance of this article would not disgrace the Spectator.

The ninth compares the enemies of phrenology to various animals. The wasps have venom enough, but nothing more than filth and trash is to be found in their cells. The butterflies flutter in the sun, but make no provision for winter, and they die when the sun withdraws its influence. The ants are industrious enough in their way, but incapable of rising from the earth and extending their views all over nature. The geese have a very solemn gait and carriage, delight in low grounds and waters, and attack all who approach them, but their hissing attacks are always laughed at. The ducks resemble the geese, but are much inferior, and so dirty as to prey on garbage: they attack with crying quack! quack! The owls are blinded by the light of the sun. The parrots say what they have learnt from others by rote, without knowing the meaning. The monkeys grin and chatter with no more sense, and are moreover very mischievous. The bears are rough and uncultivated, inhabiting the coldest and most barren regions, and despising the beauties of nature: their hug is formidable, but they may be caught and muzzled, when, after proper discipline, they form a natural alliance with the monkey, and afford pretty sport for children. The swine wallow in mud. The asses, very patient and sedate, no doubt, are, however, so obstinate, that neither blows nor kindness have the least effect upon them. The curs, though fawning enough upon occasion, are perpetually snarling and yelping at some animal nimbler than themselves, but it sometimes happens that a steed, irritated by a more than ordinary tone of impertinence in his canine foe, will lend the yelper such a salute as may lay him sprawling in the kennel, and, changing his yelp into a howl, send him home limping and lamed for life. The editors of the journal profess to know the kennel of animals well, and to have sometimes listened to their midnight howlings; in which, for the most part, they say it is impossible to distinguish any thing more than the gabbling of geese, the screeching of owls, the chattering of monkeys and parrots, the growling of bears, the grunting of hogs, the braying of asses, and the yelping of curs, and the collection is found at Blackwood's menagerie. By this article it appears that, while the Transactions are dignified and intended for grave philosophers, the Journal will be frolicsome, and castigate, without mercy, all unfair, uninformed, and impertinent opponents.

The tenth is a letter from Miss Cordelia Heartless, informing us how she explained to her inamorato that he had not her heart, but a bump in her head. It is witty enough, but would equally serve as a little quiz upon phrenology.

The eleventh is the first specimen of the application of phrenology to criticism. The piece is Macbeth, and the writer most successfully shows that Shakespeare has drawn the character, not only, as we know, in perfect uniformity with nature, but with phrenology. To give a specimen is impossible in such a continued application of phrenology to the whole feelings and conduct of Macbeth, but the harmony of the character with the phrenological view of human nature is incontestible; and, the truth of the character to nature being allowed, the harmony is no small confirmation of the truth of phrenology. Some few have thought Macbeth's character inconsistent—that a man so good in some points could never have brought himself to commit such an atrocious deed. But, if we look abroad, if we look into ourselves, we shall find great inconsistency of character. This undeniable truth is inexplicable upon all the metaphysical views of human nature; but, upon phrenology, quite explicable. Phrenologists teach, that dispositions the most opposite may exist in the mind; that in all men are certain faculties, (no matter for the name,) often, indeed, conspiring to the same end, and always intended by the Almighty for a good purpose, but frequently drawing in different directions, and, as one or the other gets the upper-hand, so will the character appear in an opposite point of view.

In the next number we are promised a phrenological review of Quentin Durward; the trials of Margaret Lindsey; Mr. Owen's new views of society; the parliamentary report on Millbank Penitentiary; and of Dugald Stewart on Milton's Garden of Eden.

The eleventh article is on the skulls of three murderers in the museum of the College of Surgeons, Dublin, and affords a good specimen of the mistakes and the unfounded reports thence arising which occur every day among persons unacquainted with, and not over friendly to, phrenology. We often hear it said that such a man's skull is any thing but accordant with his character, while, on inspection, a phrenologist finds it an admirable confirmation of his doctrine. This it appears was the case with the skulls here described. The person who showed them in the museum begged the visiter to remark that the organs of destructiveness, &c. were not larger than in ordinary men. On a careful examination, it turned out that their development of those skulls was more murderous and brutal than even that of the skulls of Gordon and Bellingham.*

The thirteenth is upon materialism and scepticism, being a sort of critique upon the Rev. T. Rennels' Pamphlet, and Somotopsychonoologia.

Most persons who rail against materialism know nothing of the subject which they presume to talk of, and not a few, we are ashamed to confess it, (and some of those are of our own profession,) appear to make a clamour in the hope of conciliating the good graces of that numerous party, many of whom mean well and who have much in their power.

Now the word materialist sometimes signifies a man who believes that the universal system has been formed by chance,

---

* The grossest mis-statements went forth to the public respecting the head of Thurtell.

and that there is no God. Such a man cannot surely exist at present. If he does, he should be pitied rather than condemned; his intellect must be deficient: the *fool* hath said in his heart there is no God. Such a man can do no harm. He cannot convince the sane; and, if he could, the laws and duties of morality would still remain written on our hearts and the voice of conscience must still be heard.

The word materialist is sometimes used to denote a man who believes in God, but thinks there is no immaterial principle in man. Suppose him right, would not the laws of God—the laws of morality written by Nature in our hearts, as St. Paul declares, be equally clear and equally imperative? And if God be pleased to reveal to us that we shall live hereafter, can the disbelief of an immaterial principle within us prevent us from believing his revelation? If it were *demonstrable philosophically* that we have an immaterial principle, and that this principle is *therefore* immortal, we may rest assured there would have been no revelation to inform us of the truth:—Christ would not have risen from the dead the first fruits of them that slept, to convince us of it. Again, what difficulty does the belief of an immaterial principle remove? The resurrection, we read, is of body as well as mind—men will rise *with their* bodies, and not only has Paley a sermon urging this, and Dr. Chalmers one in a volume just published in which he contends that the next world will be as material as the present, but our mother church has so great a respect for matter as to declare that the Almighty is invested with it in Heaven:—one of her articles asserts, that the very and eternal God ascended into Heaven, and there sits with *body*, *flesh*, and *bones*.* Away, then, with this intolerance of materialists—this invective and contempt worthy of the dark ages. We have to do with only what we observe, and neither the existence nor non-existence of an immaterial principle can be proved, nor is it of the least importance to philosophy, to morals, or to religion. Moreover, to charge phrenology with materialism is too bad. He who believes that a blow on the head will stun, is as much a materialist as he who believes in phrenology. We earnestly recommend the paper before us to the careful perusal of every one. It is written powerfully and clearly, and is worthy of a better place than a journal. We suspect, from the style, that it is by Mr. George Combe, but that is of no consequenec. We have heard some declare it should be written in letters of gold.

Of the two remaining papers, one is a short account of the Transactions, and the other a capital quiz upon those who

---

* Article IV. in conjunction with article II.

maintain the mind to be independent of the head. It is the prospectus of a high German quack in bad German English, and is very facetious.

Highly as we think of the journal, we regret that it should condescend to notice every folly and scurrility, and still more that it should occasionally notice them in the way of railing for railing. If phrenology is true, the grandeur of its truth will bear down all the fry of petty assailants, and silent contempt will often be its most efficient and dignified mode of conduct. All respectable and sound, or at least apparently reasonable, objection should receive attention and be respectfully answered; and, in a journal, new illustrations of the doctrines, such as must be daily occurring, should be set forth, and all phrenological news given. But good part of the Introductory Statement, and the whole of the Enemies of phrenology had, perhaps, been better omitted, and such papers as the Suppression of Phrenology, a Dialogue between a Philosopher of the Old School and a Phrenologist, the Spider and the Bee, the Letter from Miss Cordelia Heartless, and Donnerblitzenhausen's prospectus, should be sparingly inserted, whatever their merit, because they do not afford new facts or additional light. Still we hardly object to the number of such papers in the first volume, any more than to the Introductory Statement, the Outlines of Phrenology, and the View of some of Dr. Spurzheim's Lectures in the Transactions; because the subject may be fairly thought to be by no means familiar to the generality of readers. But future numbers should certainly consist of papers of a different description.

---

