# Errata in the Phrenological Article

**Published:** 1824-03-01

**Authors:** 


ERRATA IN THE PHRENOLOGICAL ARTICLE.

Page 864, fourth line from bottom, for *compassion* read *comparison.*
Page 874, fourth line of note, for *shall* read *should.*
Page 881, nineteenth line from top, for *truth* read *resurrection.*

